# „Er weiß es noch nicht“ – Triadische Kommunikation und ihre Tücken am Beispiel eines onkologischen Visitengesprächs

**DOI:** 10.1007/s10354-022-00924-3

**Published:** 2022-04-06

**Authors:** Bettina Baldt

**Affiliations:** Institut für Systematische Theologie und Ethik, Universität Wien, Schenkenstr. 8–10, 1010 Wien, Österreich

**Keywords:** Medizinische Kommunikation, Patientenzufriedenheit, Partizipative Gesprächsführung, Krebs, Linguistische Gesprächsanalyse, Medical communication, Physician-patient communication, Patient participation, Patient satisfation, Linguistic analysis of conversation

## Abstract

Medizinische Kommunikation ist ein wirkungsvolles Instrument ärztlichen Handelns. Aufgrund des niedrigen Stellenwerts, den medizinische Kommunikation innerhalb der Medizin und medizinischen Ausbildung immer noch einnimmt, wissen viele Ärzt:innen nicht, professionell mit diesem Instrument umzugehen. Medizinische Kommunikation so einzusetzen, dass Patient:innen gut informiert und involviert sind, ist Voraussetzung dafür, dass diese selbstbestimmt Entscheidungen treffen können. In dieser Arbeit analysiere ich ein Visitengespräch auf einer onkologischen Station gesprächsanalytisch. Auf diese Weise sollen die Tücken der ärztlichen Gesprächsführung – speziell in der triadischen Kommunikation – anhand eines Fallbeispiels aus der Praxis aufgezeigt und aus dem Transkript heraus Optimierungsvorschläge erarbeitet werden. Fehler in der Gesprächsführung, welche man in der Theorie durchaus als solche erkennt, passieren in der Praxis leider dennoch, weswegen dieses Fallbeispiel dazu einladen soll, die eigene Gesprächsführung kritisch zu reflektieren. Das Fazit enthält Anregungen für die Vorbereitung auf das Visitengespräch sowie für die Gesprächsführung während der Visite selbst.

## Abstract (en)

Medical communication is an effective instrument for the medical practice. Due to the low status that medical communication still has within medicine and medical education, many physicians do not know how to use this instrument professionally. Using medical communication in a way that patients are well informed and involved is a prerequisite for them to make self-determined decisions. In this paper I analyze a consultation in an oncology ward to show the pitfalls of medical communication, especially in triadic communication, based on a case study to work out suggestions for optimization from the transcript. Mistakes in conducting conversations, which can be recognized as such in theory, unfortunately still happen in practice, which is why this case study is intended as an invitation to critically reflect on one’s own conduct of conversations. The conclusion contains suggestions for the preparation as well as the conduct of the consultation during medical rounds.

Kommunikation zwischen Ärzt:innen und Patient:innen ist ein wichtiger Faktor für eine gelingende Interaktion und maßgeblich für die Zufriedenheit der Patient:innen [1, 2] und den Behandlungserfolg [3, 4]. Medizinische Kommunikation wurde bereits aus den Blickwinkeln unterschiedlichster Fragestellungen erforscht, wobei es sich dabei nicht nur um ein Thema der Sprachwissenschaften und verwandter Fachbereiche handelt [5, 6], sondern auch die medizinethische Relevanz deutlich hervortritt. Beauchamp und Childress formulierten als eines ihrer 4 Grundprinzipien ärztlichen Handelns die Patient:innenautonomie; Patient:innen sollen also selbstbestimmt (mit-)entscheiden können, was mit ihnen geschieht [7]. Dazu müssen Ärzt:innen ihre Patient:innen allerdings über deren Erkrankung sowie Behandlungsoptionen informieren und sie aktiv in Entscheidungsprozesse einbinden. Dafür ist eine professionelle Gesprächsführung grundlegend.

In der Sprachwissenschaft erhielten v. a. das medizinische Erstgespräch und seine Frage-Antwort-Sequenzen, wie sie beispielsweise für ein Anamnesegespräch üblich sind, viel Aufmerksamkeit [8–10]. Auch zum Visitengespräch finden sich zahlreiche Publikationen [11–13], von denen viele die Unzulänglichkeiten der Kommunikation bei Visiten zum Vorschein brachten. Beckmann und Frankel stellten fest, dass nur ein Viertel der Ärzt:innen ihre Patient:innen ausreden lässt; Unterbrechungen durch Ärzt:innen fanden durchschnittlich bereits nach 18 s statt [14]. Darüber hinaus werden 90 % der Patient:innen-Fragen in einem Visitengespräch schlecht, nicht zufriedenstellend oder nicht angemessen beantwortet [15]. Eine weitere Studie ergab, dass nur 4 von 35 Patient:innen alle ihre Anliegen ansprechen konnten und das Gespräch zufrieden verließen [16]. In einer Untersuchung von Langewitz nahm ein Visitengespräch durchschnittlich 2,5 bis 3 min in Anspruch, wovon 60 % der Redezeit auf Ärzt:innen, 30 % auf Patient:innen und 10 % auf das Pflegepersonal entfielen [17]. Auch wenn für manche Zahlen aufgrund einer mangelhaften Datenlage auf ältere Studien zurückgegriffen werden muss, finden sich auch im Jahr 2016 noch einige Kritikpunkte an der Praxis des Visitengesprächs, wobei die Kommunikation und der Informationsaustausch einen zentralen Platz einnehmen [18].

Offenbar stellt es Ärzt:innen in Visitengesprächen vor eine große Herausforderung, ihren eigenen Aufgaben (Einholen von Informationen über den Gesundheitszustand der Patient:innen, Vorantreiben der Behandlung) zeiteffizient nachzukommen, ohne die Bedürfnisse der Patient:innen (Information über Diagnose und Therapieoptionen erhalten, Einbringen eigener Anliegen) zu vernachlässigen. Optimierungsvorschläge wären ausreichend vorhanden [19, 20], dennoch belegt eine aktuellere systematische Untersuchung, dass Ärzt:innen wenig Patient:innen-involvierendes Verhalten zeigen [21]. Dieser Befund zeigt auf, dass sich nicht die Frage stellt, *ob *medizinische Kommunikation ethisch relevant ist, sondern *wie* gute Ärzt:innen mit ihren Patient:innen kommunizieren sollen, um medizinethischen Ansprüchen gerecht zu werden.

In der vorliegenden Arbeit analysiere ich ein Visitengespräch als Fallstudie medizinischer Kommunikation, wobei dieses folgende Besonderheiten aufweist: Es handelt sich dabei einerseits um eine Folgekonsultation eines chronisch kranken Patienten und andererseits sind es (primär) 2 Ärzte und 1 Patient, die am Visitengespräch beteiligt sind. Nach Grüninger bestehen Folgekonsultationen für Ärzt:innen aus Aufgaben, wie Handlungsbereitschaft und Fertigkeiten der Patient:innen zu besprechen, an der Umsetzung verfolgter (Behandlungs‑)Strategien zu arbeiten oder Unterstützung anzubieten [22]. Folgeuntersuchungen laufen daher anders ab als Erst- bzw. Anamnesegespräche, wobei außerdem unterschieden werden muss, ob es sich um eine Nachsorgeuntersuchung handelt oder um einen Termin im Rahmen einer länger andauernden Behandlung. Triadische Kommunikation wurde im medizinischen Kontext bisher beispielsweise bei Gesprächen mit Dolmetscher:innen [23] oder mit Patient:innen und deren Angehörigen erforscht [24]. Aber auch Visitengespräche, die ohne Dolmetscher:innen oder Angehörige geführt werden, spielen sich selten „nur“ zwischen einer Ärztin bzw. einem Arzt und einer Patientin bzw. einem Patienten ab, da die Visite meist von einem ganzen Team durchgeführt wird, welches sich zu gegebenem Anlass ins Gespräch einbringt. Die Untersuchung triadischer Kommunikation im Krankenhaus hat daher eine hohe Praxisrelevanz. Ziel dieser Arbeit ist es, Herausforderungen der triadischen Kommunikation beim Visitengespräch aufzuzeigen und konkrete Optimierungsvorschläge herauszuarbeiten.

## Methode

Das Visitengespräch ist Teil einer anfallenden Stichprobe, welche für ein Projekt des Wissenschaftsfonds FWF im Mai 2018 in einem österreichischen Ordensspital erhoben wurde. Aus der Stichprobe wurden 13 Visitengespräche ausgewählt, welche medizinische Entscheidungen beinhalteten. Das vorliegende Visitengespräch illustriert besonders gut die Tücken der triadischen Kommunikation, weshalb dieses für eine eigene Fallstudie herangezogen wurde, um einige wichtige Punkte der medizinischen Kommunikation zu erläutern. Die krankenhauseigene Ethikkommission genehmigte das Projekt, und die beteiligten Ärzte (zwischen 55 und 60 Jahren alt) sowie der Patient (67 Jahre alt) haben ihr schriftliches Einverständnis für die Teilnahme an der Studie gegeben. Der Patient verfügte über eine private Versicherung und war daher auf der „Sonderklasse“ untergebracht. Das Gespräch dauerte ca. 8 min, wurde mittels digitalen Audiorekorders aufgezeichnet und nach GAT-2-Konventionen für das Basistranskript verschriftet [25]. Die wichtigsten Transkriptkonventionen befinden sich im Anhang in Tab. 1.

Das gesamte Transkript wurde mittels linguistischer Gesprächsanalyse untersucht – eine Methode, um Arten interpersoneller Kommunikation sowie kommunikative Aufgaben zu rekonstruieren, Kommunikationsregeln zu identifizieren und Kommunikationspraktiken auf einer empirischen Basis zu definieren [26]. Dabei geht man sequenzanalytisch vor und untersucht die Redebeiträge der Beteiligten in Bezug auf die Sprecherwechsel, ihre interaktive Konstruktion sowie die Form der kommunikativen Handlung und deren thematischer Verknüpfung. Außerdem werden Gesprächsabschnitte und Gesprächsphasen identifiziert. In weiterer Folge werde ich einzelne, für den Gesprächsverlauf relevante Transkriptausschnitte zeigen und gesprächsanalytisch interpretieren. Aus den Ergebnissen der Analyse werden dann Optimierungsvorschläge ausgearbeitet.

## Fallstudie

Der Patient leidet an einer Krebserkrankung und wird bereits seit Jahren in der Institution betreut. Er ist daher mit dem Krankenhaus, den behandelnden Ärzt:innen sowie dem Pflegepersonal gut vertraut. Für den geplanten Krankenhausaufenthalt ist die Gabe seiner Chemotherapie angesetzt. Der Patient wurde am Morgen desselben Tages stationär aufgenommen und hatte ein Aufnahmegespräch mit einem der am Visitengespräch beteiligten Ärzte (A2). Der visitenleitende Arzt (A1) führt das Gespräch mit dem Patienten (P1) gemeinsam mit dem begleitenden Arzt (A2). Des Weiteren ohne Wortmeldung während der ausgewählten Abschnitte anwesend sind eine Gesundheits- und Krankenpflegerin, welche die Stationsleitung innehat, sowie der Stationsarzt, der kurz nach Gesprächsbeginn hinzustößt, und B.B.

### Sprecherkennzeichnungen

- A1: Visitenleitender Arzt
- A2: Begleitender Arzt
- P1: Patient

### Gesprächseröffnung

**Figure 1:**
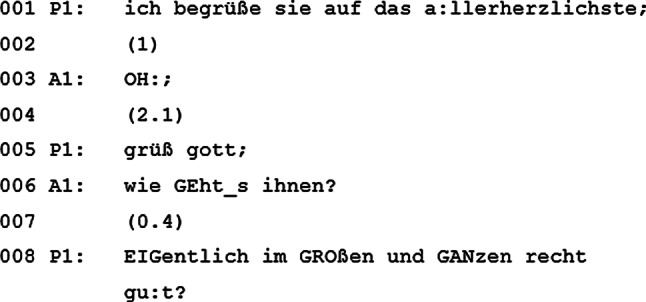

Das Visitenteam betritt das Zimmer des Patienten, woraufhin dieser das Team willkommen heißt (Abb. 1).

Der Patient eröffnet das Gespräch mit einer Begrüßung (Zeile 001). Es ist ungewöhnlich, dass der Patient das Gespräch eröffnet und nicht der visitenleitende Arzt. Dies ist ein erster Hinweis darauf, dass der Patient bereits gut mit der Institution vertraut ist. Nicht die Ärzte begrüßen ihn auf *ihrer* Station, der Patient begrüßt die Ärzte in *seinem* Zimmer. Der visitenleitende Arzt reagiert auf die Begrüßung mit einem lauten „OH:“ (Zeile 003). Diese Lautäußerung entspricht nicht der klassischen Antwort auf eine Begrüßung, weshalb der Patient seine Begrüßung reformuliert (Zeile 005). Interessant ist, dass der visitenleitende Arzt erneut nicht mit einer Gegen-Begrüßung antwortet, sondern eine Frage an den Patienten richtet (Zeile 006). Damit hebt er seine Rolle als visitenleitender Arzt hervor, da nun der Patient seine Frage beantworten „muss“. Der Patient antwortet, dass es ihm „im GROßen und GANzen EIGentlich recht gu:t“ gehe (Zeile 008).

In der Eröffnungssequenz richten sich Arzt und Patient gemeinsam aus und nutzen diese Abstimmung, um zu ermitteln, inwiefern sie miteinander kooperieren werden [27]. Obwohl der Patient zu Beginn ein wenig dominant für das Setting agiert, verhält er sich kooperativ, als der visitenleitende Arzt die Rolle des Gesprächsführers einnimmt. Der visitenleitende Arzt und der Patient tauschen sich in weiterer Folge noch detaillierter über die Verfassung des Patienten aus (Zeile 009–022).

### Eine unerwartete Wendung

**Figure 2:**
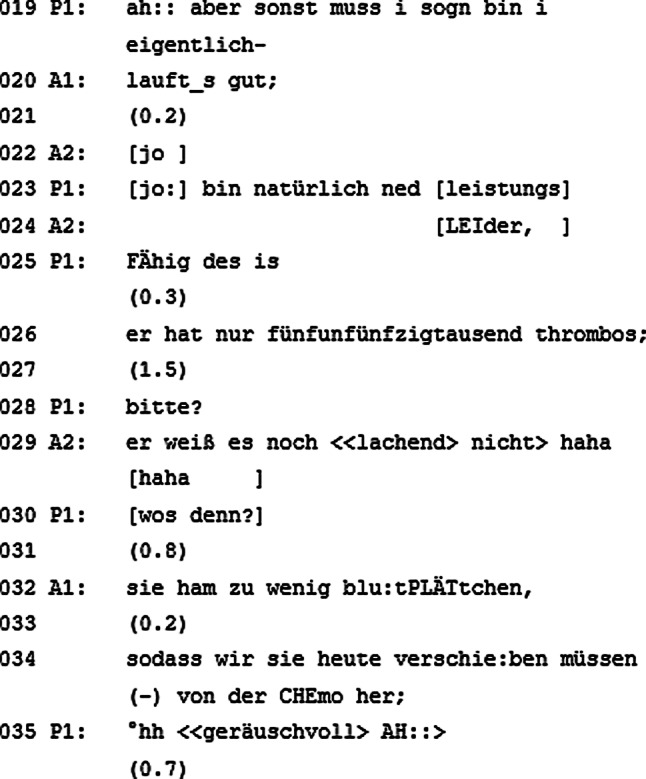

Am Beginn des Gesprächsabschnitts geht der Patient erneut auf sein Wohlbefinden aus dem vorangegangenen Gesprächsabschnitt ein, lässt seinen Satz aber unvollendet (Abb. 2, Zeile 019). Mit diesem Redebeitrag knüpft der Patient an die Frage des visitenleitenden Arztes in Zeile 006 an. Der visitenleitende Arzt beendet daraufhin den Redebeitrag des Patienten; beide Gesprächsteilnehmer zeigen sich kooperativ. Nun kommt es jedoch zu einer Überlappung zwischen dem Patienten und dem begleitenden Arzt, da beide gleichzeitig zu sprechen beginnen (Zeile 022–023). Der begleitende Arzt, welcher bisher noch nicht am Gespräch beteiligt war, verstummt wieder, als er bemerkt, dass der Patient seinerseits einen neuen Redebeitrag beginnt.

Der Patient hat sein Rederecht vorerst durchgesetzt (Zeile 023), allerdings fällt ihm der begleitende Arzt erneut ins Wort (Zeile 024). Dies ist deswegen als Unterbrechung des Patienten zu erkennen, da der begleitende Arzt mitten im Redebeitrag vom Patienten versucht, das Rederecht zu übernehmen, und die Äußerung thematisch nicht mit einem vorherigen Redebeitrag verknüpft ist. Der Patient bricht daraufhin seine Äußerung ab. Der begleitende Arzt adressiert mit seiner Unterbrechung den visitenleitenden Arzt und spricht dabei über den Patienten in der dritten Person (Zeile 026: „*er* hat nur […]“). Der begleitende Arzt informiert den visitenleitenden Arzt darüber, dass der Patient nur 55.000 Thrombozyten hat. Der Patient versteht den Einwurf des begleitenden Arztes – sei es akustisch oder inhaltlich – nicht, lässt sich dadurch aber vorerst nicht verunsichern und fragt mit einem „bitte?“ nach, um seinem Verständnis auf die Sprünge zu helfen (Zeile 028). Sein Versuch, sich wieder in das Gespräch einzubringen, aus dem er soeben (durch das Sprechen über anstatt mit ihm) ausgeschlossen wurde, gelingt nicht. Der begleitende Arzt klärt den Patienten weder darüber auf, was die von ihm eingebrachte Information für den Patienten bedeutet, noch entschuldigt er sich beim Patienten für die Unterbrechung, den Ausschluss aus dem Gespräch oder die Unhöflichkeit, in seinem Redebeitrag über ihn zu sprechen. Auch die nächste Wortmeldung ist an den visitenleitenden Arzt adressiert und dient dazu, ihn darüber aufzuklären, dass der Patient davon bis zum momentanen Zeitpunkt noch nichts wusste (Zeile 029). Der begleitende Arzt erzeugt hier mit seinem Sprechen über den Patienten ein „Gespräch im Gespräch“.

Die Frage des Patienten bleibt indes unbeantwortet, was ihn dazu veranlasst, diese in anderen Worten zu wiederholen (Zeile 030). An dieser Stelle wäre eine Videoaufnahme von Vorteil, um zu überprüfen, ob die nonverbale Kommunikation Aufschluss darüber gibt, wen der Patient mit seinen Fragen in Zeile 028 und 030 adressiert. Ob nonverbal adressiert oder nicht, der visitenleitende Arzt antwortet beim zweiten Versuch auf die Frage des Patienten, indem er die Worte des begleitenden Arztes aus Zeile 026 paraphrasiert und in eine leichter verständliche Sprache übersetzt (Zeile 032: „sie ham zu wenig blu:tPLÄTtchen“). Anschließend erklärt der visitenleitende Arzt dem Patienten, welche Folgen die geringe Thrombozytenanzahl für seine Behandlung hat (Zeile 034: „sodass wir sie heute verschie:ben müssen (–) von der CHEmo her;“).

**Figure 3:**
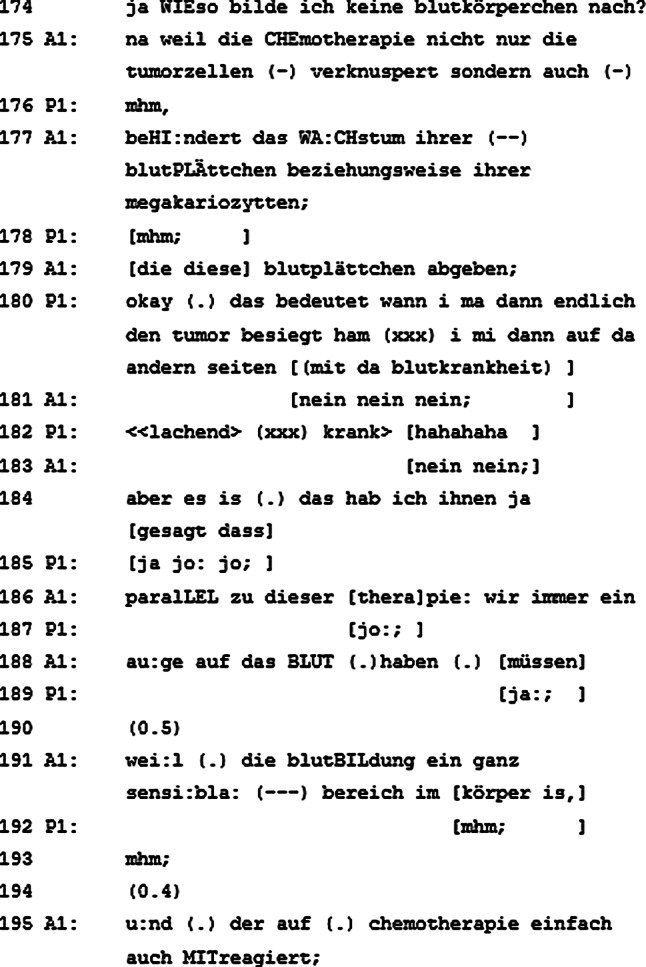

Nach dieser Sequenz wird gemeinsam von Ärzten, Pflegeperson und Patient ein Ersatztermin für die Chemotherapie gesucht. Dabei wird auf den geplanten Urlaub des Patienten Rücksicht genommen, über welchen der begleitende Arzt schon Bescheid weiß, der visitenleitende Arzt aber noch nicht. Abermals wird das Gespräch dadurch gestört, dass nicht alle am Gespräch Beteiligten denselben Informationsstand haben. Nachdem das Missverständnis aufgeklärt ist und sich alle Beteiligten auf eine Woche geeinigt haben, in der die Chemotherapie nachgeholt werden soll, erkundigt sich der Patient nach seinem „Gesamtbild“, woraufhin die Ärzte darlegen, dass sie mit den anderen Werten des Patienten sehr zufrieden sind. Der Patient führt die überraschende Besserung auf eine Änderung bei der vergangenen Chemotherapie zurück, welche es laut den Ärzten aber nicht gab. Nachdem die Sache (zumindest zur Zufriedenheit der Ärzte) geklärt ist, wird ein konkreter Termin für die verschobene Chemotherapie vereinbart, den alle Beteiligten bestätigen. Nun stellt der Patient die Frage, die ihn wohl schon beschäftigt, seit der visitenleitende Arzt ihm erklärt hat, dass er zu wenige Thrombozyten für die Chemotherapie hat (Abb. 3, Zeile 174, thematische Verknüpfung zu Zeilen 032–034).

### Die Frage im Hinterkopf

Die Frage in Zeile 174 könnte direkt an die Erklärung des visitenleitenden Arztes in Zeile 034 anschließen. Es ist daher naheliegend, dass die Erklärung des visitenleitenden Arztes für den Patienten nicht ausreichend war. In den Zeilen 175 bis 179 gibt der visitenleitende Arzt ausführlich Antwort auf die Frage des Patienten. Besonders ins Auge fällt die Wortwahl „verknuspern“, die im Zusammenhang mit einer Tumorerkrankung recht ungewöhnlich ist und bagatellisierend wirkt. Es fehlen an dieser Stelle die Hinweise darauf, ob der visitenleitende Arzt der Erkrankung mit Humor begegnen will oder sich eine Sprache zunutze macht, die sonst eher bei Kindern Anwendung findet. Die Pause unmittelbar vor dem Wort „verknuspern“ deutet jedenfalls darauf hin, dass der visitenleitende Arzt die Formulierung bewusst wählt.

Der Patient ratifiziert die Erklärung des visitenleitenden Arztes (Zeile 180), geht dann jedoch zu einer Interpretation der Ausführungen des visitenleitenden Arztes über und äußert seine Sorgen in Bezug auf die ungewollten Konsequenzen, welche die Chemotherapie mit sich bringen könnte. Diese Interpretation hält der visitenleitende Arzt für unzulässig und begleitet diese mit mehrmaligen Verneinungen. Der Rest des Redebeitrags des Patienten geht in seinem eigenen Lachen unter – eine Reaktion auf die vehemente Negierung seiner Äußerung durch den visitenleitenden Arzt. Mit seinem Lachen könnte der Patient zum Ausdruck bringen, dass er seine „Schwarzmalerei“ in Bezug auf die negativen Folgen der Chemotherapie nicht ernst gemeint hat. Der Optimismus bzw. die Hoffnung des Patienten zeigt sich auch in seiner Äußerung in Zeile 180: „[…] wann i ma dann endlich den tumor besiegt ham […].“

**Figure 4:**
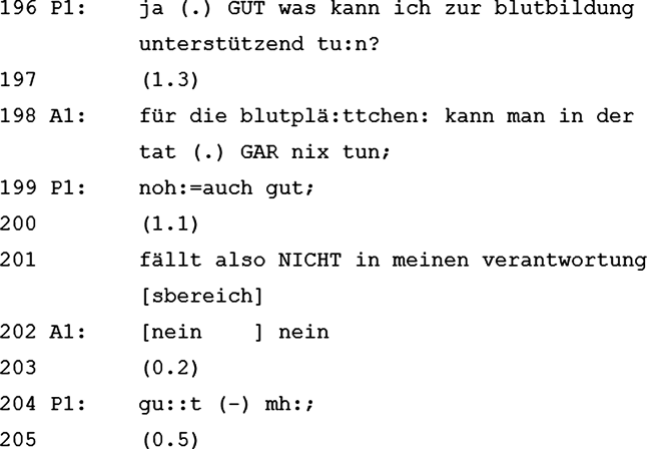

Von Zeile 184 bis 195 führt der visitenleitende Arzt noch einmal aus, dass er den Patienten bereits auf die möglichen Nebenwirkungen der Chemotherapie hingewiesen hat und dass sie die Blutbildung im Auge behalten müssen, da dies ein sensibler Bereich des Körpers ist. Nachdem der visitenleitende Arzt die Frage des Patienten zu dessen Zufriedenheit beantwortet hat, stellt der Patient eine weitere Frage (Abb. 4, Zeile 196).

### Was kann der Patient tun?

Der Patient ergreift erneut die Initiative und erkundigt sich beim visitenleitenden Arzt, wie er zur Blutbildung beitragen kann. Der visitenleitende Arzt antwortet, dass der Patient zur (Nach‑)Bildung der Blutplättchen nichts beitragen könne. Dies konnotiert der Patient mit einem „noh:=auch gut;“ (Zeile 199) und zeigt sich erleichtert, dass er dafür keine Verantwortung trägt (Zeile 201). Wie zu Beginn des Gesprächs begibt sich der Patient in diesem Abschnitt wieder in eine ungewöhnliche Rolle: Er hat die Gesprächsführung inne, ergreift oft das Wort, vertieft ein Thema und eröffnet ein Anschlussthema. Auch wenn der Patient selbst keine Möglichkeiten hat, auf die Blutbildung einzuwirken, löst er mit seiner Frage in Zeile 196 doch eine Modifizierung der Chemotherapie aus (Zeilen 206–227). Der visitenleitende Arzt kündigt an, bei der nächsten Chemotherapie eine Substanz zu reduzieren, welche die Blutplättchen besonders angreift.

**Figure 5:**
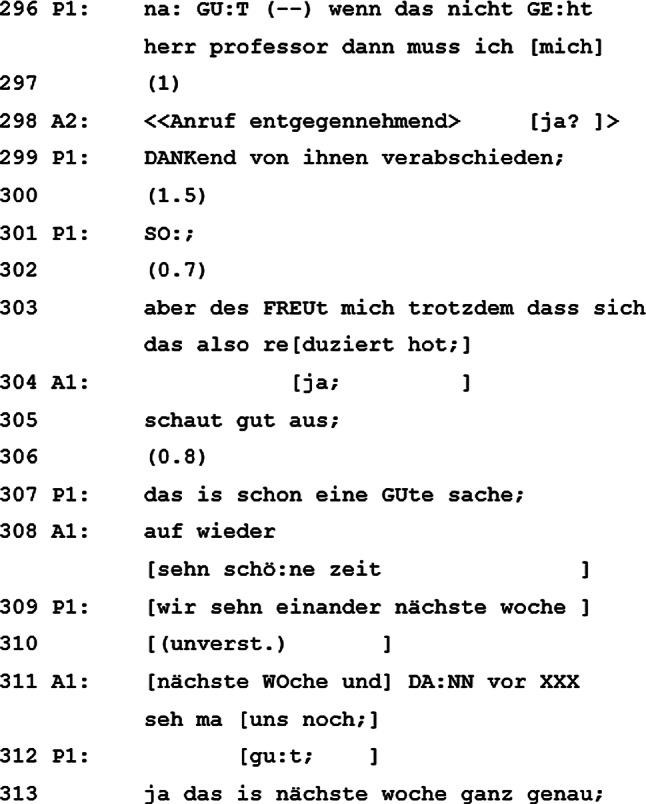

Nach diesem Gesprächsabschnitt liefert der visitenleitende Arzt noch eine Zusammenfassung dessen, was bisher besprochen wurde (Zeilen 228–268), und kommt dadurch wieder auf den geplanten Urlaub des Patienten zu sprechen. Der Patient freut sich, dass sich die Verschiebung der Chemotherapie gut mit dem Urlaub vereinbaren lässt. Der visitenleitende Arzt offenbart einen persönlichen Bezug zum Urlaubsort des Patienten und empfiehlt diesem, viel zu gehen. Der Patient wiegelt ab und meint, er sei dafür zu schwach. Der visitenleitende Arzt gibt weitere Tipps, wie es der Patient anstellen könnte, sich mehr zu bewegen, der Patient bleibt jedoch unverbindlich und leitet das Ende des Gesprächs ein (Abb. 5, Zeilen 296, 299); eine weitere unübliche Handlung für einen Patienten in einem Visitengespräch.

### Gesprächsende

Da er heute keine Chemotherapie bekommen würde, muss er sich dankend vom „Herrn Professor“ verabschieden. Es wirkt, als „verleihe“ der Patient dem visitenleitenden Arzt hier einen noch höheren Titel, um diesen milde zu stimmen, weil der Patient untypischerweise das Gesprächsende einleitet. Zusätzlich wird das Gespräch durch einen Anruf gestört, welchen der begleitende Arzt annimmt und sich damit aus dem Gespräch zurückzieht. Es folgt eine Verabschiedungssequenz zwischen visitenleitendem Arzt und Patienten (Zeilen 303–313), in welcher der Patient noch einmal hervorhebt, wie sehr er sich freut, dass sich die anderen Werte reduziert haben. Obwohl der Patient nicht erhält, wofür er eigentlich gekommen ist – nämlich seine Chemotherapie – beendet er das Gespräch positiv gestimmt. Bezeichnend für das gesamte Gespräch behält der Patient das letzte Wort.

### Zusammenfassung

Bevor ich dazu übergehe aufzuzeigen, welche Schlüsse aus diesem Visitengespräch zu ziehen sind, fasse ich hier noch einmal kurz die relevanten Interaktionsphänomene zusammen, um einen guten Überblick über die Analyse zu geben:

Der Patient eröffnet das Gespräch, visitenleitender Arzt und Patient stellen sich aufeinander ein. Es folgt eine Unterbrechung durch den begleitenden Arzt, welcher eine neue Information einbringt, die klar macht, dass der Patient an diesem Tag keine Chemotherapie erhalten wird (Neuausrichtung des Gesprächs). Während der begleitende Arzt dem visitenleitenden Arzt diese neue Information mitteilt, wird der Patient aus dem Gespräch ausgeschlossen (Gespräch im Gespräch). Der Patient versucht 2‑mal, sich wieder ins Gespräch hineinzureklamieren; beim zweiten Versuch antwortet ihm der visitenleitende Arzt. Da die Chemotherapie heute nicht mehr stattfinden kann, wird ein neuer Termin überlegt. Wieder ist der begleitende Arzt im Gegensatz zum visitenleitenden Arzt bereits über die Urlaubspläne des Patienten informiert (Missverständnis). Nachdem der neue Termin fixiert ist, kehrt der Patient zum Problem der zu geringen Blutplättchen zurück (Frage im Hinterkopf). Er fordert damit eine ausführlichere Antwort ein als der visitenleitende Arzt am Beginn des Gesprächs gegeben hat. Der Patient erkundigt sich weiter, was er tun kann, um die Bildung der Blutplättchen zu unterstützen (Patient übernimmt Rolle des Gesprächsführers). Nachdem der visitenleitende Arzt noch einmal alle relevanten Inhalte zusammengefasst hat und für den Patienten alle Punkte geklärt sind, leitet der Patient das Gesprächsende ein.

## Fazit

### Wie könnte das vorliegende Visitengespräch optimiert werden?

Allein der Umstand, dass die beiden Ärzte nicht über denselben Informationsstand verfügen, bewirkt eine komplette Neuausrichtung des Gesprächs und verursacht ein weiteres Missverständnis; beides wäre einfach zu vermeiden gewesen. Im besten Fall haben alle denselben Informationsstand, wobei der Informationsaustausch zwischen Visitenteam und Patient:in durchaus während der Visiten stattfinden kann und soll. Um das Gespräch möglichst effizient zu gestalten und das Sprechen über Patient:innen zu vermeiden, wäre es jedoch von Vorteil, wenn sich das Visitenteam bereits vor dem Betreten des Patient:innen-Zimmers bespricht, wie das auch Weber und Kolleg:innen vorschlagen: Es wird im Visitenteam geklärt, welche 3 bis 5 Themen bei der Visite im Fokus stehen sollen, was die Ziele für die Patientin bzw. den Patienten sind und wer welche Themen anspricht [13].

Lässt es sich einmal nicht vermeiden, Kolleg:innen während der Visite über Neuigkeiten zu informieren, sollte dies mit einer metakommunikativen Äußerung begleitet werden und nach Möglichkeit die Patientin bzw. den Patienten als Adressat miteinschließen. Dadurch sollen Zwiegespräche in einer triadischen Konstellation und daraus resultierende Kommunikationsgefälle vermieden werden. Im vorliegenden Visitengespräch könnte das wie folgt umgesetzt werden: „Entschuldigen Sie bitte die Unterbrechung, aber bevor Sie das Gespräch weiter fortsetzen, muss ich Sie noch über eine wichtige Information in Kenntnis setzen. Herr P, Sie haben zurzeit nur 55.000 Blutplättchen. Dieser Wert ist zu niedrig, weshalb Sie heute doch keine Chemotherapie bekommen können. Dr. XY, welche weiteren Schritte würden Sie vorschlagen?“ Nachdem die nächsten Schritte besprochen sind, sollte der Patient ausreichend Gelegenheit erhalten, Fragen dazu zu stellen.

### Ist eine Optimierung wirklich notwendig?

Im vorliegenden Visitengespräch werden augenscheinlich alle Fragen des Patienten, wenn auch nicht auf Anhieb, beantwortet und seine Nachfrage bewirkt im Folgenden die Veränderung der Zusammensetzung seiner Chemotherapie. Warum sollte das Visitengespräch also optimiert werden? Der Patient in diesem Fallbeispiel ist bereits gut mit der Institution und den behandelnden Ärzten vertraut, was sich beispielsweise schon in der Begrüßungssequenz zeigt. Er bekommt Antworten auf seine Fragen, weil er beharrlich bleibt, wenn er nicht gehört wird und sich des Öfteren wieder ins Gespräch hinein reklamiert.

Es ist anzunehmen, dass andere Patient:innen nicht so durchsetzungsfähig sind und sich im Kontext der institutionellen Kommunikation nicht trauen, derartig zu agieren. Joseph-Williams et al. zeigten in ihrer systematischen Übersichtsarbeit, dass viele Patient:innen immer noch glauben, sich passiv verhalten zu müssen, um nicht als „schwierige Patient:innen“ zu gelten [28]. Dazu gehört auch, keine Fragen zu stellen und keine zweite Meinung einzuholen. Es ist daher besonders wichtig, dass Ärzt:innen Patient:innen bewusst die Möglichkeit geben, Fragen zu stellen und Unklarheiten zu äußern.

Eine Möglichkeit, um sicherzustellen, dass Patient:innen relevante Informationen verstanden haben, ist die Teach-Back-Methode. Nach der Erklärung des Arztes bzw. der Ärztin bittet diese:r beispielsweise: „Ich möchte sichergehen, dass ich es gut erklärt habe. Können Sie mir sagen, wie die Anzahl der Blutplättchen mit Ihrer Chemotherapie zusammenhängt?“ Ärzt:innen können so das Verständnis der Patient:innen direkt überprüfen und bei Missverständnissen ihre Erklärung nachbessern. Eine Übersichtsarbeit zur Methode zeigte darüber hinaus positive Auswirkungen auf die Patient:innenzufriedenheit, das Krankheitsmanagement und -wissen der Patient:innen sowie deren gesundheitsbezogene Lebensqualität [29]. Außerdem ist das Verständnis relevanter Informationen von Patient:innen nicht nur grundlegend für ein selbstbestimmtes Mitgestalten im Sinne der Patient:innenautonomie, sondern ebenso für eine gemeinsame Entscheidungsfindung im Sinne eines „shared decision making“, bei dem Ärzt:innen und Patient:innen Entscheidungen auch gemeinsam verantworten.

## Anhang

**Table 1:**

[ ]	Überlappungen und Simultansprechen
=	Schneller, unmittelbarer Anschluss neuer Redebeiträge
°h / h°	Ein- bzw. Ausatmen von ca. 0,2–0,5 s Dauer
°hh / hh°	Ein- bzw. Ausatmen von ca. 0,5–0,8 s Dauer
(.)	Mikropause, geschätzt, bis ca. 0,2 s Dauer
(-)	Kurze geschätzte Pause von ca. 0,2–0,5 s Dauer
(--)	Mittlere geschätzte Pause von ca. 0,5–0,8 s Dauer
(0.5)	Gemessene Pause von 0,5 s Dauer
:	Dehnung, Längung, um ca. 0,2–0,5 s
::	Dehnung, Längung, um ca. 0,5–0,8 s
und_äh	Verschleifungen innerhalb von Einheiten
akZENT	Fokusakzent
?	Hoch steigende Intonation
,	Mittel steigende Intonation
–	Gleichbleibende Intonation
;	Mittel fallende Intonation
.	Tief fallende Intonation
(unverst.)	Unverständliche Passage
